# Two-stage surgical strategy for extensive craniofacial fibrous dysplasia with cerebral compression

**DOI:** 10.1007/s00701-025-06438-2

**Published:** 2025-02-12

**Authors:** Ludovica Pasquini, Chandrima Biswas, Marcus Zachariah, Daniel M. Prevedello

**Affiliations:** 1 Department of Neurological Surgery, The Ohio State University Wexner Medical Center, Columbus, OH USA; 2 Department of Neurological Surgery, Neurosurgical Medical Clinic, Sharp Memorial Hospital, San Diego, CA USA

**Keywords:** Cerebral compression, Craniofacial fibrous dysplasia, McCune-albright syndrome, Surgical resection

## Abstract

**Background:**

The management of extensive craniofacial fibrous dysplasia requires balancing the extent of resection with the perioperative morbidity and complications.

**Method:**

The authors describe a case involving the resection of extensive craniofacial fibrous dysplasia performed in two stages. The first surgery aims on removing most of the lesion and planning for bony reconstruction, while the second stage focuses to complete disease removal and implant a custom-made prosthesis.

**Conclusion:**

This case highlights the benefits of a two-stage surgical approach in reducing morbidity compared to a single extensive surgery while achieving excellent disease resection and functional outcomes.

**Supplementary Information:**

The online version contains supplementary material available at 10.1007/s00701-025-06438-2.

## Manuscript

### Relevant surgical anatomy

Craniofacial fibrous dysplasia (FD) is a bone disorder that involves several contiguous bones of the facial skeleton, most notably the frontal bone, those forming the orbit and the frontal sinus.

### Frontal, sphenoid, and ethmoid bones relationships

The frontal, sphenoid, and ethmoid bones create a complex anatomical interface in the craniofacial region, influencing cranial integrity and separation from the orbital, nasal and intracranial structures. The frontal bone connects to the ethmoid via the cribriform plate, which contains foramina for olfactory nerve fibers. Posteriorly, the perpendicular plate of the ethmoid bone articulates with the sphenoid bone through the sphenoidal process. Additionally, the anterior sphenoid connects to the frontal bone at the inferior wall of the frontal sinus, while the greater wings of the sphenoid extend laterally, interacting with orbital structures [4].

### Frontal sinus

The frontal sinus (FS) is a paired cavity in the frontal bone. Absent at birth, the FS becomes visible by age 4 and continue to expand until approximately age 16 [3]. The horizontal orbital plate represents the thinnest FS wall. Inferiorly, it narrows towards the frontal ostium in the infundibulum, which, along with the recess, forms the drainage pathway. Lined with mucosa, the FS humidifies air and secretes mucus, which drains into the nasal cavity via the frontonasal duct [8].

## Description of the technique

**Fig. 1:**
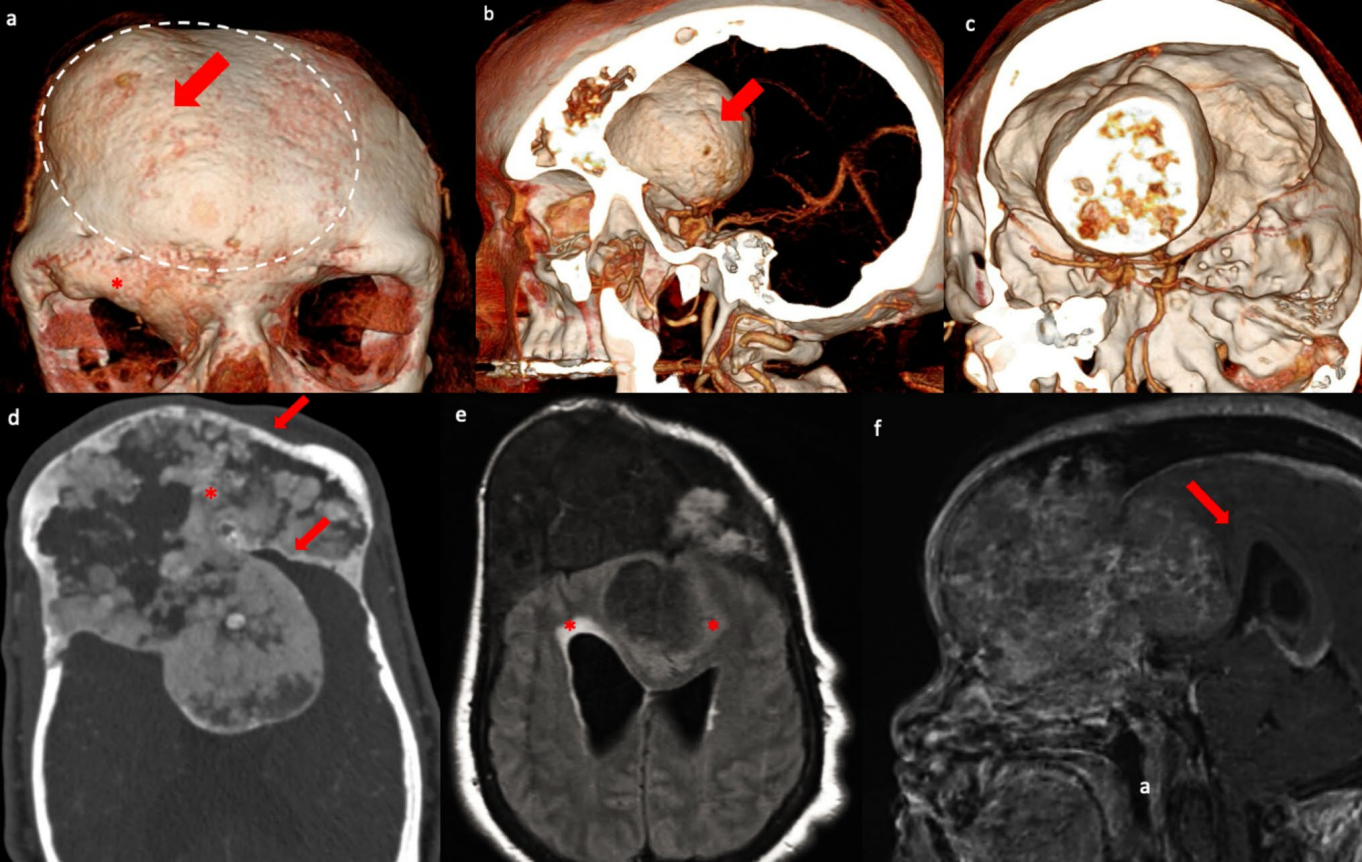
Preoperative imaging work-up: fine-cut computed tomography (CT), CT angiography (CTA) and brain magnetic resonance image (MRI). **a** Coronal 3D-reconstruction from CT scan demonstrated a large bony deformity in the right portion of the anterior calvarium (arrow) and the right superior orbital rim (asterisk). The white dashed circle highlights the planned bifrontal craniectomy. **b** Sagittal 3D-reconstruction from CTA showed a large intracranial pedunculated mass (arrow) within the anterior and middle cranial fossae. **c** Coronal 3D-reconstruction from CTA revealed the anatomical relationship between the intracranial mass and the major cerebral arteries. CTA did not identify any significant arterial feeders to embolize prior to surgery. **d** Axial CT scan with bone window settings revealed complete obliteration of the frontal sinus due to the bony growth (asterisk), along with disease extension into its anterior and posterior wall (arrows). **e** Axial T2-FLAIR MRI highlighted hyperintense signals in the periventricular area and around the intracranial lesion (asterisks). The lesion’s size had remained stable in comparison with the previous imaging; however, the FLAIR signal change represented a new finding. **f** Sagittal T1-postcontrast MRI demonstrated an extensive, irregularly enhancing lesion involving the frontal, sphenoidal, and ethmoidal regions, causing severe cerebral mass effect, particularly on the corpus callosum (arrow)

A 46-year-old woman with McCune-Albright syndrome presented with frontal lobe symptoms and facial deformity. The symptoms gradually progressed over three years, becoming severely debilitating in the past 12 months. Imaging revealed extensive craniofacial FD involving the frontal, ethmoid, sphenoid bones, as well as a large intracranial mass in the anterior and middle cranial fossae, causing cerebral mass effect (Fig. 1).

## First surgery

**Fig. 2:**
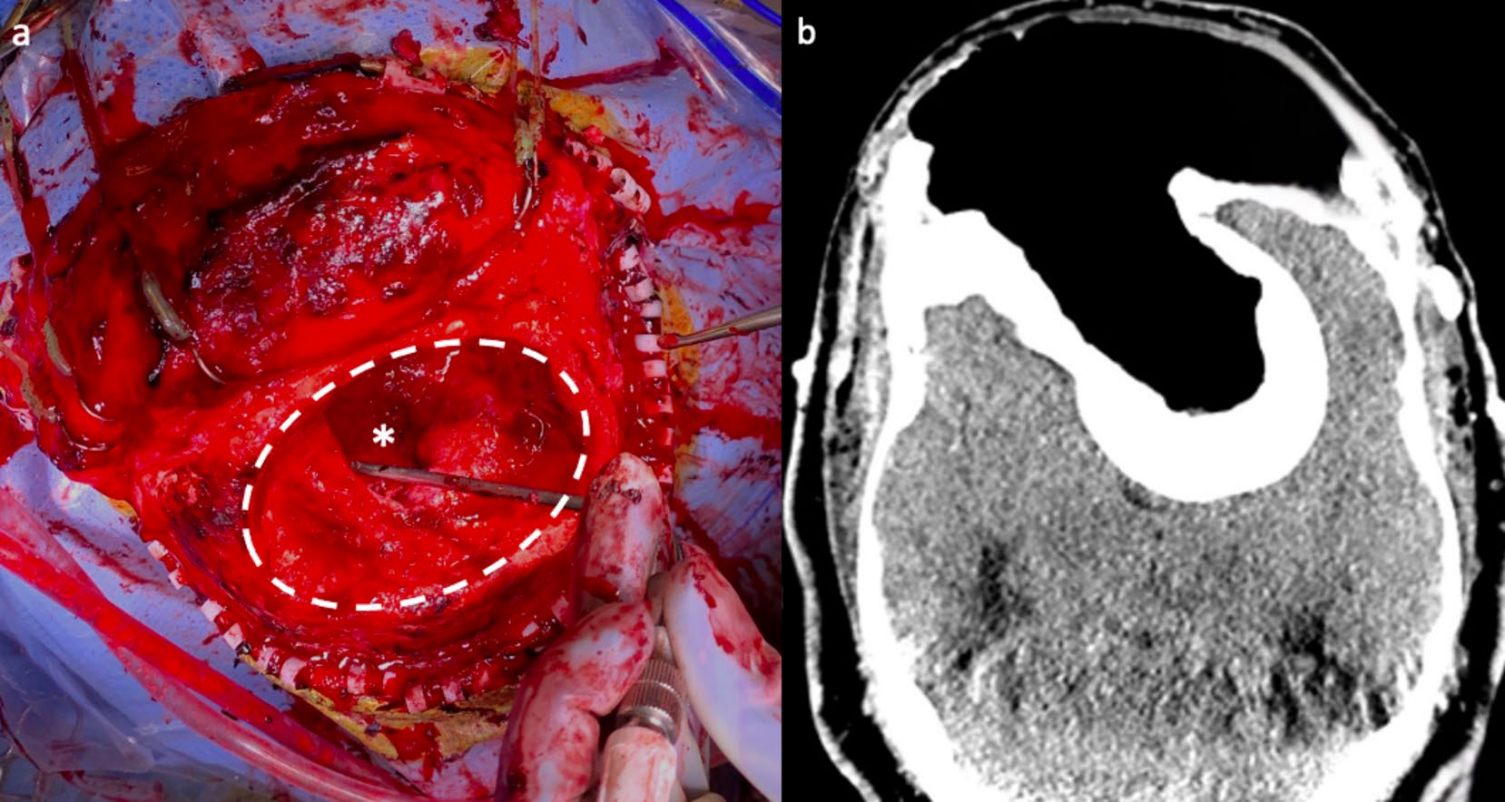
First surgery images. **a** Intraoperative picture showing the surgical cavity after the bifrontal craniectomy (dashed circle), drilling of the superficial lesion, and partial debulking of the intracranial mass (asterisk). **b** Axial CT scan after the first surgery demonstrated the removal of the superficial disease and intra-tumoral debulking of the pedunculated mass

Electrophysiological monitoring of somatosensory evoked potentials (SSEPs) and neuronavigation were established. After general anesthesia, the patient was positioned supine with the head secured in a neutral position using a Mayfield support. A bicoronal skin incision was made, and after mobilizing the scalp to the frontal prominence just above the orbital rim, two surgeons operated simultaneously on either side of the head to reduce surgical time. Using two sets of drills, a bifrontal craniectomy was performed, providing access to the superficial lesion encased in the severely dilated FS. The lesion was carefully removed, and the right superior orbital prominence was recontoured to restore facial symmetry. Neuronavigation helped define the limits of the bilateral bony growth, allowing for precise drilling of the tumor portion near the superior sagittal sinus (SSS), while preserving it. At this point, drilling of the pedunculated mass was initiated, remaining entirely intra-tumoral without exposing the dura. At the end of the debulking, three large oval-shaped, grayish-brown, irregularly bony fragments—measuring 6.8 × 4.4 × 2.3 cm, 8.6 × 4.5 × 2.5 cm and 5.8 × 4.8 × 1.7 cm—were removed. The surgical cavity was packed with Gel-Foam sponges after ensuring hemostasis. The galea and skin were meticulously closed over the bony defect (Fig. 2a).

### Postoperative course

The patient was initially transported to the Intensive Care Unit, where she remained neurologically and hemodynamically stable. The postoperative period was free of any complications and follow-up confirmed proper incision healing. A fine-cut CT scan was performed to create a 3D custom-made polyetheretherketone (PEEK) cranioplasty based on the margins of the superficial bony defect (Fig. 2b).

## Second surgery

The second stage was performed two months later to allow the patient to full recover from the first procedure.

**Fig. 3:**
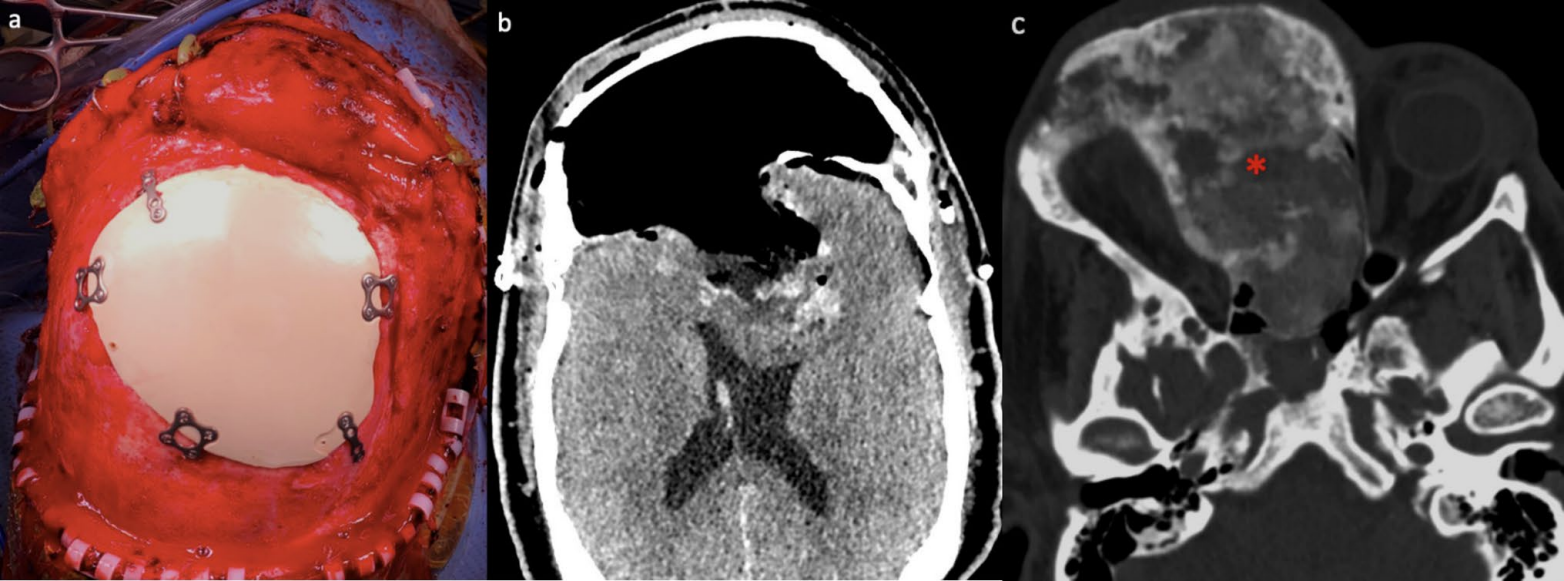
Second surgery images. **a** Intraoperative picture demonstrated the placement of the custom-made PEEK cranioplasty, fixed with multiple titanium miniplates, perfectly fitting the cranial defect. **b** Axial CT scan after the second surgery showed the removal of the extensive FD lesion, brain decompression, and reconstruction of the anterior calvarium. **c** Axial CT scan with bone settings window highlighted the residual disease

The patient was positioned as in the previous procedure, and the prior incision was reopened, tilting the flap forward to expose the bone around the craniectomy. Burr holes were made laterally to inspect the dura, which appeared intact. At this point, we thinned the bone within the entire cavity, and carefully lifted it. A 6.6 × 5.1 × 3.6 cm irregular lobulated portion of grayish-pink bone was extracted. Under microscopic visualization, we finalized the resection in the deep portion, where the lesion was firmly attached to the dura and, at one point, had invaded the pia of the frontal lobes, requiring meticulous dissection for complete removal. We reconstructed the anterior calvarium with the patient-specific implant, which fit the superficial defect perfectly (Fig. 3a), with no issues arising from skin retraction during placement.

This case involved extensive disease affecting the facial bones and anterior skull base. In similar cases, surgery should be tailored to the patient’s symptoms, avoiding removal of the skull base disease in the absence of functional deficits [7]. As the patient exhibited symptoms of cerebral compression, our goal was to remove as much disease as possible to relieve pressure on the brain. Residual disease was left in the ethmoid region to avoid the complex challenges of skull base reconstruction and potential complications.

## Postoperative course

After the two-stage surgery, the patient improved significantly, and postoperative CT scan confirmed a gross-total resection of the intracranial mass and frontal bone vault, with cerebral decompression (Fig. 3b). Pathological analysis confirmed the FD diagnosis.

**Fig. 4:**
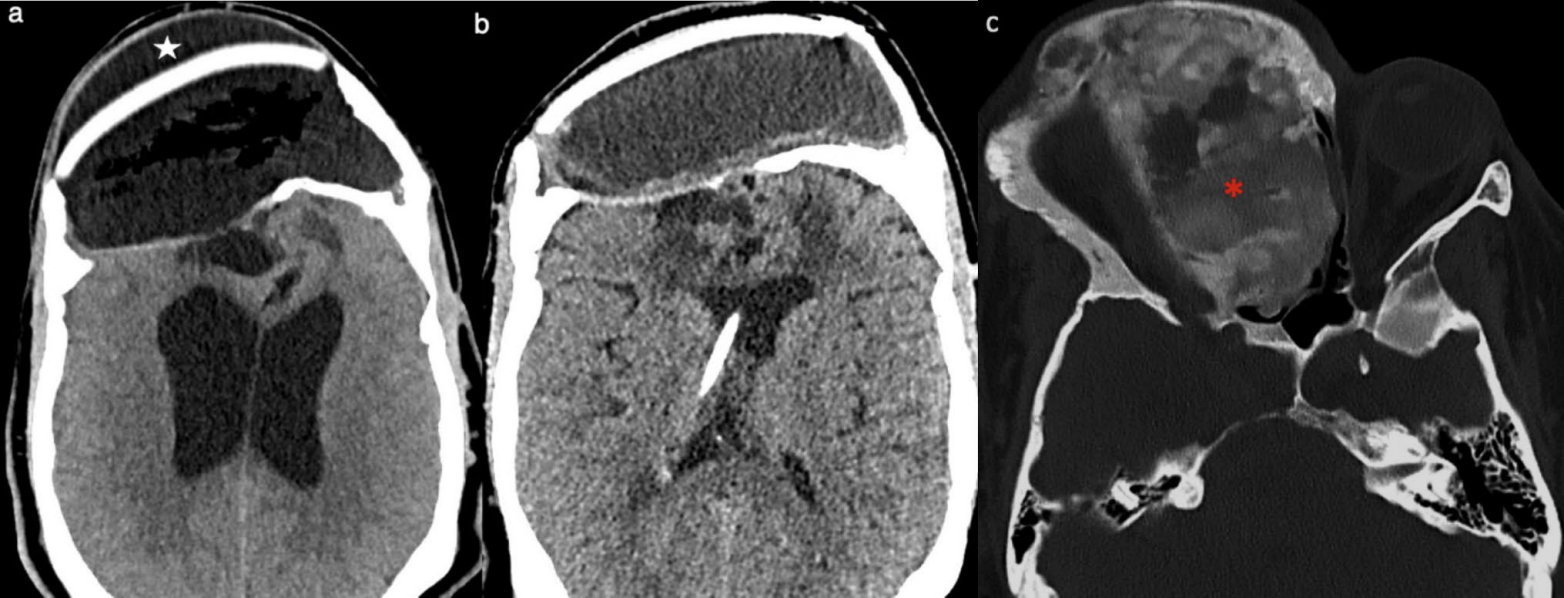
Follow-up images. **a** Axial CT scan 5-months after surgery demonstrated dilatation of the lateral ventricles and pseudomeningocele. The white star highlights the cerebrospinal fluid collection around the cranioplasty. The patient underwent a ventriculoperitoneal shunt placement. **b** Axial CT scan after 4-years showed significant decrease in ventricular size, and stable postoperative changes. **c** Axial CT scan with bone window settings revealed that the untreated disease remained stable

At the 5-month follow-up, the patient experienced headache, and CT showed pseudomeningocele and hydrocephalus, treated initially conservatively but later requiring a ventriculoperitoneal shunt. After 4 years, the patient remained neurologically stable, with no disease recurrence or symptoms from the untreated area (Fig. 4).

## Indication

McCune-Albright syndrome represents the most severe form of FD, characterized by the replacement of normal bone with irregular fibro-connective tissue, along with pigmented cutaneous lesions and/or endocrinological abnormalities [5]. Craniofacial FD typically manifests in childhood as a slow-growing, painless mass causing facial asymmetry, with progression during adolescence [6]. Surgical intervention is the primary treatment. When feasible, demolitive and reconstructive procedures are preferred over less aggressive techniques, such as bone curettage, as they significantly reduce recurrence rates [1, 2, 10]. Although craniofacial FD often stabilize in adulthood, our patient began experiencing symptoms of brain compression in adulthood, with associated FLAIR signal changes that were not evident in the previous imaging. Surgical treatment was therefore recommended to address both functional and aesthetic concerns. The two-stage surgical approach was selected based on the lesion’s size and the gradual progression of symptoms in a stable, non-acute clinical condition. This approach allowed for reduced risks associated with a single complex procedure, improved cerebral decompression, and the opportunity to plan a custom-made cranioplasty.

### Limitations

In two-stage surgery, the patient consequently undergoes two anesthesiological procedures. However, this approach reduces the amount of tissue and blood vessels involved, minimizing bleeding and morbidity associated, while allowing time for the patient to stabilize between surgeries.

#### Potential complications and measures taken to avoid them:

1. Protect the edematous brain by preserving the subarachnoid/pial plane and cortical veins, avoiding excessive brain retraction, and using neuronavigation to maintain SSS integrity.
2. Avoid damaging the ependymal barrier during deep lesion removal with magnification and careful dissection. However, in extensive cases, cerebrospinal fluid leak may occur, potentially worsened by ex-vacuo hydrocephalus. Conservative treatment is an option, but a CSF shunt may be considered if necessary.
3. In the second stage, preserve the superficial bony perimeter for proper cranioplasty fitting and to prevent cosmetic deformity.
4. Preoperative planning and two-stage approach reduce the risk of optic and vascular injury, whit the first stage focusing on intra-tumoral debulking and the second completing resection without breaching the pial plane.

### Specific information for the patient

Patients should be aware that the primary goal for FD/MAS treatment is to preserve function, reduce morbidity, and improve aesthetics, not to cure the disease. Regular follow-up, including endocrinological management, remains crucial for monitoring progression and ensuring stability [9].

## Supplementary Information

Below is the link to the electronic supplementary material.

ESM 1 (MP4 442 MB )

## Data Availability

No datasets were generated or analysed during the current study.

## Abbreviations

FD: Fibrous dysplasia
FS: Frontal sinus
PEEK: Polyetheretherketone

## References

[1] Andejani D, Mrad MA (2018) Enlargement of Frontal Sinus, Case Report. Aesthetic Plast Surg 42(4):1013–1018. 10.1007/s00266-018-1106-1 29492672 10.1007/s00266-018-1106-1

[2] Guatta e R, Scolozzi P (2018) Bone Recontouring by Guided Surgical Navigation Integrating ‘Mirroring’ Computational Planning in the Management of Severe Fronto-orbital Asymmetry in Fibrous Dysplasia. J Neurol Surg Part Cent Eur Neurosurg 79(2): 181–185 10.1055/s-0037-1615286 10.1055/s-0037-1615286 29294508

[3] Hartley I, Zhadina M, Collins MT, Boyce AM (May 2019) Fibrous dysplasia of bone and McCune–Albright Syndrome: a bench to Bedside Review. Calcif Tissue Int 104(5):517–529. 10.1007/s00223-019-00550-z 10.1007/s00223-019-00550-z PMC6541017 31037426

[4] Javaid MK, Boyce A, Appelman-Dijkstra N, Ong J, Defabianis P, Offiah A, Arunde P, Shaw N, Dal Pos V, Underhil A, Portero D, Heral L, Heegaard AM, Masi L, Monsell F, Stanton R, Dijkstra PDS, Brandi ML, Chapurlat R, Hamdy NAT, Collins MT (2019) Best practice management guidelines for fibrous dysplasia/McCune-Albright syndrome: a consensus statement from the FD/MAS international consortium. Orphanet J Rare Dis 14:139. 10.1186/s13023-019-1102-9 31196103 10.1186/s13023-019-1102-9 PMC6567644

[5] Kim DY (2023) Current concepts of craniofacial fibrous dysplasia: pathophysiology and treatment. Arch Craniofac Surg 24(2):41–51. 10.7181/acfs.2023.00101 37150524 10.7181/acfs.2023.00101 PMC10165234

[6] Lee J et al (May 2012) Clinical guidelines for the management of craniofacial fibrous dysplasia. Orphanet J Rare Dis 7. 10.1186/1750-1172-7-S1-S2 10.1186/1750-1172-7-S1-S2 PMC3359960 22640797

[7] McLaughlin RB, Rehl RM, Lanza DC (2001) Clinically relevant frontal sinus anatomy and physiology. Otolaryngol Clin North Am 34(1):1–22. 10.1016/S0030-6665(05)70291-7 11344058 10.1016/s0030-6665(05)70291-7

[8] Seiden AM, Stankiewicz JA (1998) Frontal sinus surgery: the state of the art. Am J Otolaryngol 19(3):183–193. 10.1016/s0196-0709(98)90086-2 9617931 10.1016/s0196-0709(98)90086-2

[9] Szymczuk V, Taylor J, Boyce eAM (Apr. 2023) Craniofacial Fibrous Dysplasia: clinical and therapeutic implications. Curr Osteoporos Rep 21(2):147–153. 10.1007/s11914-023-00779-6 10.1007/s11914-023-00779-6 PMC11087144 36849642

[10] Valentini V, Cassoni A, Marianetti TM, Terenzi V, Fadda MT, Iannetti eG (Feb. 2009) Craniomaxillofacial Fibrous Dysplasia: conservative treatment or radical surgery? A retrospective study on 68 patients. Plast. Reconstr Surg 123(2):653. 10.1097/PRS.0b013e318196bbbe 10.1097/PRS.0b013e318196bbbe 19182626

